# Effect of EGFR-TKI retreatment following chemotherapy for advanced non-small cell lung cancer patients who underwent EGFR-TKI

**DOI:** 10.7497/j.issn.2095-3941.2014.04.006

**Published:** 2014-12

**Authors:** Guo-Hao Xia, Yun Zeng, Ying Fang, Shao-Rong Yu, Li Wang, Mei-Qi Shi, Wei-Li Sun, Xin-En Huang, Jia Chen, Ji-Feng Feng

**Affiliations:** Department of Medical Oncology, Jiangsu Cancer Hospital, Nanjing 210009, China

**Keywords:** Non-small cell lung cancer (NSCLC), epidermal growth factor receptor tyrosine kinase inhibitor (EGFR-TKI), erlotinib, gefitinib, chemotherapy, acquired resistance

## Abstract

**Objective:**

Non-small cell lung cancer (NSCLC) patients with epidermal growth factor receptor (EGFR)-activating mutations have higher response rate and more prolonged survival following treatment with single-agent EGFR tyrosine kinase inhibitor (EGFR-TKI) compared with patients with wild-type EGFR. However, all patients treated with reversible inhibitors develop acquired resistance over time. The mechanisms of resistance are complicated. The lack of established therapeutic options for patients after a failed EGFR-TKI treatment poses a great challenge to physicians in managing this group of lung cancer patients. This study evaluates the influence of EGFR-TKI retreatment following chemotherapy after failure of initial EGFR-TKI within at least 6 months on NSCLC patients.

**Methods:**

The data of 27 patients who experienced treatment failure from their initial use of EGFR-TKI within at least 6 months were analyzed. After chemotherapy, the patients were retreated with EGFR-TKI (gefitinib 250 mg qd or erlotinib 150 mg qd), and the tumor progression was observed. The patients were assessed for adverse events and response to therapy. Targeted tumor lesions were assessed with CT scan.

**Results:**

Of the 27 patients who received EGFR-TKI retreatment, 1 (3.7%) patient was observed in complete response (CR), 8 (29.6%) patients in partial response (PR), 14 (51.9%) patients in stable disease (SD), and 4 (14.8%) patients in progressive disease (PD). The disease control rate (DCR) was 85.2% (95% CI: 62%-94%). The median progression-free survival (mPFS) was 6 months (95% CI: 1-29). Of the 13 patients who received the same EGFR-TKI, 1 patient in CR, 3 patients in PR, 8 patients in SD, and 2 patients in PD were observed. The DCR was 84.6%, and the mPFS was 5 months. Of the 14 patients who received another EGFR-TKI, no patient in CR, 6 patients in PR, 6 patients in SD, and 2 patients in PD were observed. The DCR was 85.7%, and the mPFS was 9.5 months. Significant difference was found between the two groups in PFS but not in response rate or DCR.

**Conclusion:**

Retreatment of EGFR-TKIs can be considered an option after failure of chemotherapy for patients who were previously controlled by EGFR-TKI treatment.

## Introduction

Epidermal growth factor receptor tyrosine kinase inhibitor (EGFR-TKI) has become an indispensable treatment for advanced non-small cell lung cancer (NSCLC). Based on ISEL, BR21, and other prospective clinical study results, EGFR-TKI is recommended to be a 2- or 3-frontier treatment program for advanced NSCLC. The study on IPASS^1^ shows that the effective rate of Gefitinib is 71.2% and 1.1% for EGFR and non-EGFR gene mutations, respectively. A study on WJTOG3405^2^ showed that the median progression-free survival (mPFS) of patients with EGFR gene mutations was evidently longer when treated with gefitinib compared with cisplatin plus docetaxel chemotherapy (9.2 *vs*. 6.3 months). A study on NEJ002^3^ showed that Gefitinib treatment of patients with EGFR gene mutations took up to 10.8 months. However, treatment with carboplatin and paclitaxel chemotherapy was only 5.4 months (HR, 0.30; *P*=0.001). Based on this result, the NCCN Guidelines state that advanced NSCLC patients with EGFR gene mutations prioritize EGFR-TKI when choosing a treatment. Initially, EGFR-TKI was effective in patient treatment. However, development of drug resistance resulted in unsuccessful treatments. At present, a standard treatment program does not exist. Thus, EGFR-TKI retreatment has become a research hotspot. Since September 2010, scholars have been focusing on the clinical study on EGFR-TKI retreatment after chemotherapy when NSCLC patients acquired drug resistance in the advanced stages of the initial EGFR-TKI treatment.

## Materials and methods

### Clinical data

#### Case selection criteria

(I) Age ≥18; (II) expected life is at least 12 weeks; (III) Eastern Oncology Group score of 0-2 for physical condition; (IV) the case is stage IV NSCLC diagnosed through histopathology or cytology; (V) cases are resistant to drugs after they were treated with EGFR-TKI for over 6 months, and then treated with chemotherapy for 2-4 cycles; (VI) on the time of selection, medication was stopped for at least 3 months after initial administration of EGFR-TKI; (VII) a measurable tumor based on RESIST 1.1 is observed (can be scanned by magnetic resonance imaging or computed tomography imaging technique in one dimension at least, and recorded as ≥10 mm); and (VIII) have signed an Informed Consent meeting as required by ICH-GCP guidelines. This study was approved by the ethics committee at Jiangsu Cancer Hospital.

#### EGFR genetic test

From September of 2010 to February of 2014, inspection and analysis of EGFR gene mutations were conducted on the 27 stage IV cases diagnosed through pathology or postoperative recurrence and metastasis. Tumor specimens were obtained from surgical specimens, biopsy specimens, and puncture specimens. Direct sequencing and ARMS method were used to inspect for gene mutations. Inspection was conducted before EGFR-TKI retreatment.

#### Determination of sample size

Simon’s two-stage MiniMax was used to estimate sample size. Suppose that target disease control rate (DCR) of 30% is P1, and the lowest efficacy is 80%, and, allowable missed follow-up rate is 10%. A total of 23 patients are grouped. The minimal number of event in the first test stage is one, that is, critical value into second stage is *r*=1. Rejecting the null hypothesis, the number of events is at least five, namely critical value *r*=5.

#### Diagnosis criteria

Stage IV or postoperative recurrent and metastatic NSCLC is diagnosed through histopathology or cytology by adopting the Lung and Pleura Tumor Histologic Classification Revised Proposal issued by WHO in 2004. International Staging of Lung Cancer (7th Edition) issued by the Union for International Cancer Control, and International Association for the Study of Lung Cancer in 2009 were adopted to identify the stages of lung cancer. Acquired resistance was defined as disease progression despite EGFR-TKI treatment for at least 1 month with clinical benefits [complete response/stable disease/partial response (CR/PR/SD) more than 6 months].

### Methods

#### Treatment methods

Patients received chemotherapy, including pemetrexed, gemcitabine, paclitaxel albumin, and docetaxel with or without platinum (cisplatin, carboplatin, nedaplatin). EGFR-TKI retreatment was employed when chemotherapy was unsuccessful. The patients who were initially treated with EGFR-TKI for at least 12 months were given the same TKI for retreatment, whereas those who were initially treated with EGFR-TKI for at least 6 months were given another kind of EGFR-TKI for retreatment; dosage of gefitinib (AstraZeneca Company) was 250 mg qd, or dosage of erlotinib (Roche Company) was 150 mg qd, till the progress of disease.

#### Evaluation indicator

RECIST was used for evaluation. Efficacy was evaluated after the first month of medication. Subsequent evaluations were carried out every 2 months. Safety evaluation was carried out every 2 weeks. Evaluation of adverse events was based on NCI-CTC3.0 standards for grades 0-IV. Incidence was calculated.

#### Final indicator

DCR: the number of response cases + the number of stable cases (CR + PR + SD)/the number of cases which can be evaluated.

PFS is defined as from the day into the group to the day when disease progression was first observed (based on imaging and expressed in months). For patients who died because of other causes before disease progression, PFS was calculated from the day into the group to the day of death (in months).

Safety indicators include the degree and incidence of adverse events.

### Statistical analysis

SPSS 17.0 statistical software was used for statistical analysis. Comparison between DCR and RR was conducted by direct calculation of probability (Fisher’s exact probability). Log-rank test was used to compare PFS. *P* values <0.05 were considered to be statistically significant.

## Results

### EGFR genetic test

A total of 27 NSCLC patients who were in advanced stages or postoperative recurrent and metastatic were treated with EGFR-TKI for at least 6 months. The patients then underwent EGFR-TKI retreatment after chemotherapy.

### Baseline characteristics of patients

Clinical and pathological features of all patients, including age, gender, cell types, smoker or non-smoker, gene mutation, and imaging material, are shown in **Table 1**. A non-smoker was defined as a person who smoked less than 100 cigarettes in his lifetime.

**Table 1:** Demographic characteristics and summary of prior therapy for NSCLC

Characteristics	Value (%)
Age (years)	
<65	10 (37)
≥65	17 (63)
Sex	
Female	12 (44.4)
Male	15 (55.6)
ECOG performance status	
1	20 (74)
2	7 (26)
Histology	
Adenocarcinoma	27 (100)
Smoking history	
Ex-smoker (quit >1 year before diagnosis)	3 (11.1)
Non-smoker	24 (88.9)
No. of prior chemotherapy regimens (except gefitinib/erlotinib)	
2	13 (48.2)
3	8 (29.6)
4	6 (22.2)
Best response to initial gefitinib/erlotinib therapy	
Complete response	1 (3.7)
Partial response	15 (55.6)
Stable disease	11 (40.7)
Types of progression to initial gefitinib/erlotinib therapy	
Local progression	8 (29.6)
Systemic progression	17 (63)
Both	2 (7.4)
Time to progression to initial gefitinib/erlotinib therapy (months)	
Median, 95% CI	19 (6.5-35)
6-12	7 (26)
≥12	20 (74)
Time from initial diagnosis to gefitinib/erlotinib retreatment (months)	
Median, 95% CI	25.5 (9.1-37.6)
12-24	12 (44.4)
≥24	15 (55.6)

ECOG, Eastern Cooperative Oncology Group; NSCLC, non-small cell lung cancer; CI, conﬁdence interval.

### Initial efficacy of EGFR-TKI

The patients included one CR case, 15 PR cases, and 11 SD cases. mPFS was 19 months. mPFS of the same-drug group was 20 months (range, 15-36 months), whereas mPFS of the different-drug group was 11 months (range, 6-36 months). EGFR-TKI was used as the first-line treatment for four cases (14.8%), and as second-line and above treatment for remaining cases.

### Efficacy of chemotherapy after resistance to EGFR-TKI

Pemetrexed + cisplatin 4 cases, gemcitabine + cisplatin 2 cases, paclitaxel albumin + cisplatin 2 cases, pemetrexed + carboplatin 3 cases, gemcitabine + carboplatin 1 case, pemetrexed + nedaplatin 3 cases, paclitaxel albumin + carboplatin 2 cases, gemcitabine 1 case, docetaxel 2 cases, pemetrexed 3 cases, paclitaxel albumin 3 cases. Median chemotherapy cycle was three. One patient had CR (3.7%), 4 had PR (14.8%), 10 had SD (37%), and 12 had PD (44.4%). DCR was 55.5%; mPFS was 4 months.

### Retreatment with EGFR-TKI and its efficacy

Thirteen cases were treated with the same EGFR-TKI (same-drug group, gefitinib for 9 cases and erlotinib for 4 cases), and 14 cases were treated with another EGFR-TKI (different-drug group, gefitinib for 10 cases instead of erlotinib; gefitinib for 2 cases instead of icotinib; and erlotinib for 1 case instead of gefitinib). The total effect was as follows: 3.7% for CR (1 case), 29.6% for PR (8 cases), 51.9% for SD (14 cases), and 14.8% for PD (4 cases). RR was 33.3%. DCR was 85.2% (95% CI: 62%-94%). mPFS for EGFR-TKI was 6 months. In the same-drug group, 1 case had CR (7.6%), 2 cases had PR (15.4%), and 8 cases had SD (61.5%); RR was 23%. There were 2 cases in PD (15.4%), DCR was 84.6%, and mPFS was 5 months. In the different-drug group, there were no CR cases, 6 cases in PR (42.8%), 6 cases in SD (42.8%), and 2 cases in PD (14.3%). RR was 42.8%, mPFS was 9.5 months, and DCR was 85.7%. Precise probability was used to calculate DCR of both groups. When *P*>0.05, it means that there is no significant difference. When *P*<0.05, there is a significant difference (Logrank Test) between PFS of both groups. Reused PFS of the different-drug group was evidently longer than that of same-drug group. The proportion of reused PFS to initially used mPFS, in the same-drug group was 25% (5/20), whereas it was 86% (9.5/11) in the different-drug group. Reused PFS of the same TKI was less than PFS initially used [42.8% (6/14)]. PFS by using another EGFR-TKI was greater than that initially used.

### Analysis of EGFR gene status and efficacy

Among the 27 patients, 23 underwent EGFR genetic testing, in which, 19 were mature types; 1 case was wild type, and 3 cases were unclear. Among the 19 EGFR gene mutation cases, 11 were exon 19 mature, 7 were exon 21 mature, and 1 was synchronous mature of exon 19 and exon 21. The mature patients were treated with EGFR-TKI. Efficacies were as follows: 1 case for CR (5.26%), 5 cases for PR (26.3%), and 13 cases for SD (68.4%). DCR was 100%. One wild type case underwent EGFR-TKI retreatment and the efficacy was PD.

### Effect of the initial efficacy of EGFR-TKI on retreatment with EGFR-TKI

For one patient whose initial efficacy using EGFR-TKI was CR, the efficacy of retreatment of EGFR-TKI was CR. Among the 15 patients whose initial efficacy of EGFR-TKI was PR, 5 showed efficacy of retreatment of EGFR-TKI of PR, 8 patients had SD, and 2 cases had PD. RR was 33.3% and DCR was 86.7%. Among the 11 patients whose initial efficacy of EGFR-TKI was SD, 3 cases were PR after retreatment of EGFR-TKI, 6 cases were SD, and 2 cases were PD. RR was 18.2% and DCR was 81.8%. For patients whose initial efficacy of EGFR-TKI was PR and SD, precise probability method was used for calculations. No significant difference was observed in DCR and RR by retreatment of EGFR-TKI, *P*>0.05. This result indicates that patients whose initial efficacy was PR and SD will experience the same efficacy in retreatment.

**Figure 1:**
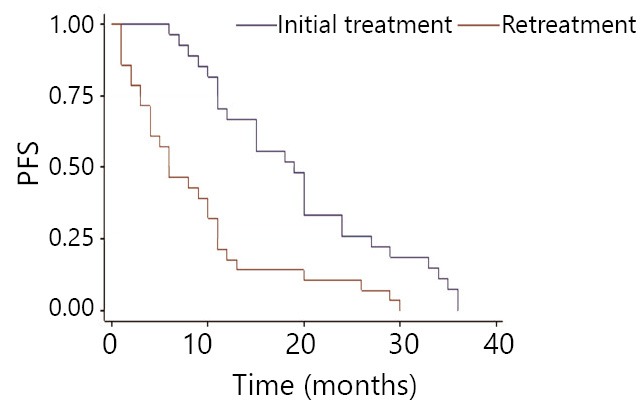
Comparison of EGFR-TKI initial treatment and retreatment in progression-free survival.

**Figure 2:**
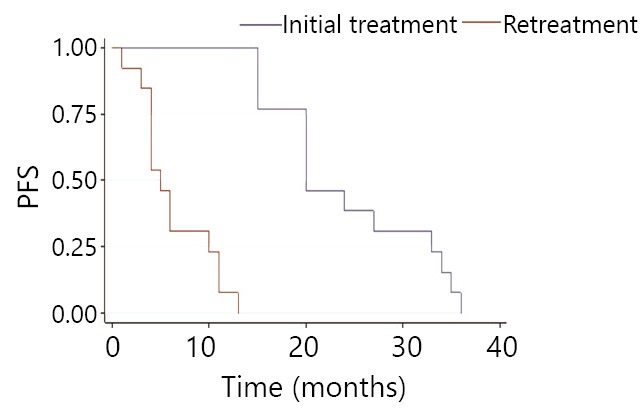
Comparison of the same drug group’s EGFR-TKI initial treatment and retreatment in progression-free survival.

**Figure 3:**
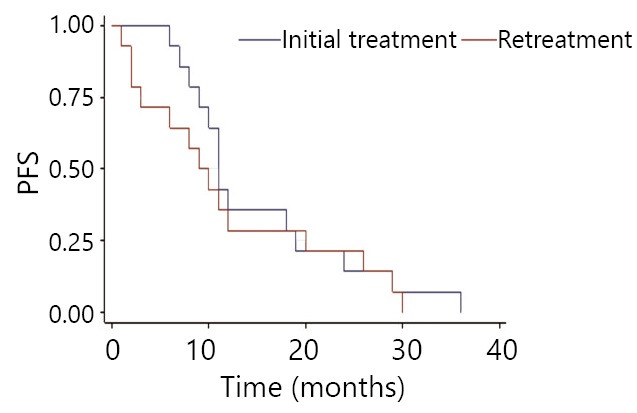
Comparison of the different drug group’s EGFR-TKI initial treatment and retreatment in progression-free survival.

**Figure 4:**
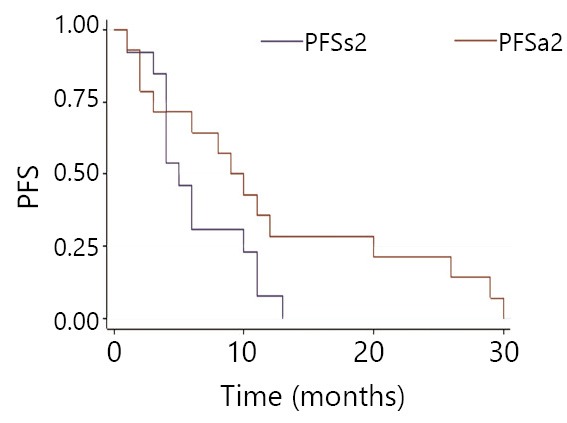
Comparison of EGFR-TKI retreatment in the disease-free survival with the same drug group (PFSs2) and the different drug group (PFSa2).

Initial mPFS is 19 months, with 20 months in the same-drug group, and 11 months in the different-drug group. Thus, a significant difference exists between these two groups (*P*<0.05). This result indicates that initial mPFS in the same-drug group using EGFR-TKI was evidently longer than that in the different-drug group. The mPFS by reusing EGFR-TKI was 6 months, 5 months in the same-drug group, and 9.5 months in the different-drug group. Thus, a significant difference exists between the two groups (*P*<0.05). This result indicates that the mPFS by reusing EGFR-TKI in the different-drug group was evidently longer than that in the same-drug group. Moreover, the PFS of reusing the same EGFR-TKI was less than initial PFS, which was 42.8% (6/14). PFS of reusing another EGFR-TKI was more than initial PFS. The proportion of mPFS of retreatment to initial mPFS was 25% (5/20) for the same-drug group, whereas it was 86% (9.5/11) in the different-drug group (**Figures 1****,****2****,****3****,****4**). The interval of two applications of EGFR-TKI was 4-15 months. The median was 7 months.

### Adverse reaction

The primary adverse reaction was level 1 or level 2 rash. The incidence was 26%. Two patients suffered level 2 rash. The secondary adverse reaction was level 1 or level 2 diarrhea. Three patients suffered level 2 diarrhea. The adverse reactions were relieved after treatment.

## Discussion

All advanced NSCLC patients treated with EGFR-TKIs acquire resistance, and no standard treatment for such patients has been established yet. To date, a considerable number of retrospective studies and one prospective study with a small sample size have reported that drug resistance was acquired after EGFR-TKIs were used to treat advanced NSCLC for a period of time. However, EGFR-TKIs retreatment was still effective to an extent; DCR was approximately 8.7%-75% and the mPFS was 1.7-6 months^4^^-^^8^. The difference in efficacy was evidently large. The treatment was unstable. Several researchers assume that patients whose PFSs of initial EGFR-TKI were greater than 5 months and above, and whose EGFR-TKI treatment interval was greater than 2 months, will have positive EGFR-TKI retreatment results. The present study evaluated the effects of EGFR-TKI retreatment following chemotherapy and unsuccessful initial EGFR-TKI treatment for at least 6 months on NSCLC patients. A considerable number of patients can obtain disease control. RR was 33.3%, DCR was 85.2%, and mPFS was 6 months. The efficacy obtained is better than results of previous studies^9^^-^^11^. Saito *et al*.^9^ evaluated the effect of erlotinib after initial treatment with gefitinib for at least 6 months on 21 lung cancer patients. There were 2 patients in PR, 9% RR, 6 cases (29%) in SD, and DCR was 38%, which was lower than the result of the present study. Notably, the patients in the previous study did not receive chemotherapy before EGFR-TKI retreatment. Oh^10^ studied the effect of gefitnib retreatment following chemotherapy and unsuccessful initial gefitinib treatment on 23 NSCLC patients. PR and DCR were 21.7% (5 cases) and 65.2% (15 cases), respectively. Efficacy of treatment slightly improved. However, results of the present study are better. In this study, 13 cases were re-treated with the same EGFR-TKI, whereas 14 cases were re-treated with a different EGFR-TKI. PFS of retreatment in the different-drug group was evidently longer than that in the same-drug group. The ratio of retreatment mPFS to initial mPFS was 25% (5/20) in the same-drug group, whereas it was 86% (9.5/11) in the different-drug group. Retreatment PFS using the same TKI is less than the initial PFS, 42.8% (6/14). Retreatment PFS using a different EGFR-TKI was higher than the initial PFS. This result indicates that retreatment with a different EGFR-TKI has a higher efficacy than retreatment with the same EGFR-TKI.

The efficacy of initial EGFR-TKI is PR. It is same as the efficacy of SD patients with EGFR-TKI retreatment but different from the results reported previously^8^.

Retreatment with EGFR-TKI is critical in the long-term survival of advanced NSCLC patients (3-5 years)^9^.

The possible mechanisms behind the positive effects of EGFR–TKI retreatment are as follows: (I) Tumor cell heterogeneity. In the presence of resistance, some tumor cells retain their original sensitive mutant and can still clone using the EGFR pathway^10^. (II) Re-selective effect of treatment augments tumor cell heterogeneity. Tumor cells do not express resistance mechanisms after drug treatment. Cells themselves generate a particular expression because of their variation or evolution. After drugs, such as EGFR-TKI, kill sensitive tumor cells, drug-resistant tumor cells are selected in the treatment. After that, drugs with completely different mechanisms, such as in chemotherapy, may kill the remaining tumor cells from the previous treatment^11^. Drugs, such as those in chemotherapy, act as selective agents. Original drugs (original TKI) can play the treatment effect. (III) Some research studies show that mutant EGFR gene is changed after chemotherapy. In general, percentage of mutant genes decreases. Before and after chemotherapy, 70% of patients retain EGFR mutant genes. Thirty percent of patients change from negative to positive or from positive to negative. This variation may be one of effective mechanisms of retreatment with EGFR-TKI. (IV) Drug holiday phenomenon. Long-term treatment of drugs with extensive dosage causes adverse reactions. When medication is stopped for a period of time, small doses of the same drug yields positive results. The sensitivity of drugs is recovered. Resistance is temporary. Levodopa is used for Parkinson’s disease in a similar way. A study showed that after metastatic NSCLC patients were treated with EGFR-TKIs (6 cases with gefitinib and 7 cases with erlotinib), the disease is progressed. Mediation was stopped for 3 weeks. There were no anti-tumor treatments during this period. Then, EGFR-TKI was used for retreatment. Out of 10 patients, 7 cases were stable and tumor sizes of 8 patients decreased^12^. Another study revealed that after tumor cells with EGFRL858R-sensitive mutation were treated with EGFR-TKI, T790M\PIK3A resistance mutation occurred. Resistance mutation disappeared when EGFR-TKI treatment ceased for a period of time^13^. For patients who received chemotherapy and stopped EGFR-TKIs treatment a longer period, the probability that resistance will be lost is higher. (V) A clinical study showed that the resistance to cytotoxic drugs was related to the EGFR pathway^14^. Activity of the EGFR pathway may increase sensitivity of EGFR-TKI^15^^,^^16^. Some cytotoxic drugs can induce gefitnib sensitivity of NSCLC cells by improving EGFR phosphorylation levels^17^. Chemotherapy in between two treatments of gefitinib may decrease the proportion of tumor cells that are resistant to gefitinib. (VI) A clinical study showed that erlotinib was also effective on gefitinib non-advantageous population. Plasma concentrations of erlotinib were markedly higher than that of gefitinib^18^^,^^19^. Half-maximal inhibitory concentration (IC_50_) of erlotinib was lower than the IC_50_ of gefitinib^20^. Therefore, erlotinib may be an effective treatment for patients resistant to gefitinib.

This study shows that retreatment with a different EGFR-TKI has higher efficacy than retreatment with same EGFR-TKI. However, a retrospective study made by Tang *et al.*^21^ showed that retreatment with a different EGFR-TKI has the same efficacy as retreatment with the same EGFR-TKI. The differences in results suggest that a randomized controlled clinical study is imperative.

In conclusion, among advanced NSCLC patients who are retreated with EGFR-TKI following chemotherapy and an unsuccessful initial EGFR-TKI for a long time (≥6 months) because of acquired resistance, a considerable part of patients can regain disease control. For patients who are successfully treated with TKI for 6-12 months, using a different EGFR-TKI has better efficacy than using the same EGFR-TKI.
